# Supplementary material for: Eye activity tracks task-relevant structures during speech and auditory sequence perception
Source: Nat Commun. 2018 Dec 18;9:5374. doi: 10.1038/s41467-018-07773-y (PMC6299078; doi:10.1038/s41467-018-07773-y)
Supplement: Supplementary file 1 — Suppelementary Information [file 41467_2018_7773_MOESM1_ESM.pdf]

Supplementary information

Eye Activity Tracks Task-Relevant Structures during Speech and Auditory Sequence Perception

Jin, Zou, Zhou & Ding

1. **Supplementary Figures**

2. **Supplementary Tables**

3. **Supplementary Methods**

4. **Supplementary Notes**

1. Supplementary Figures

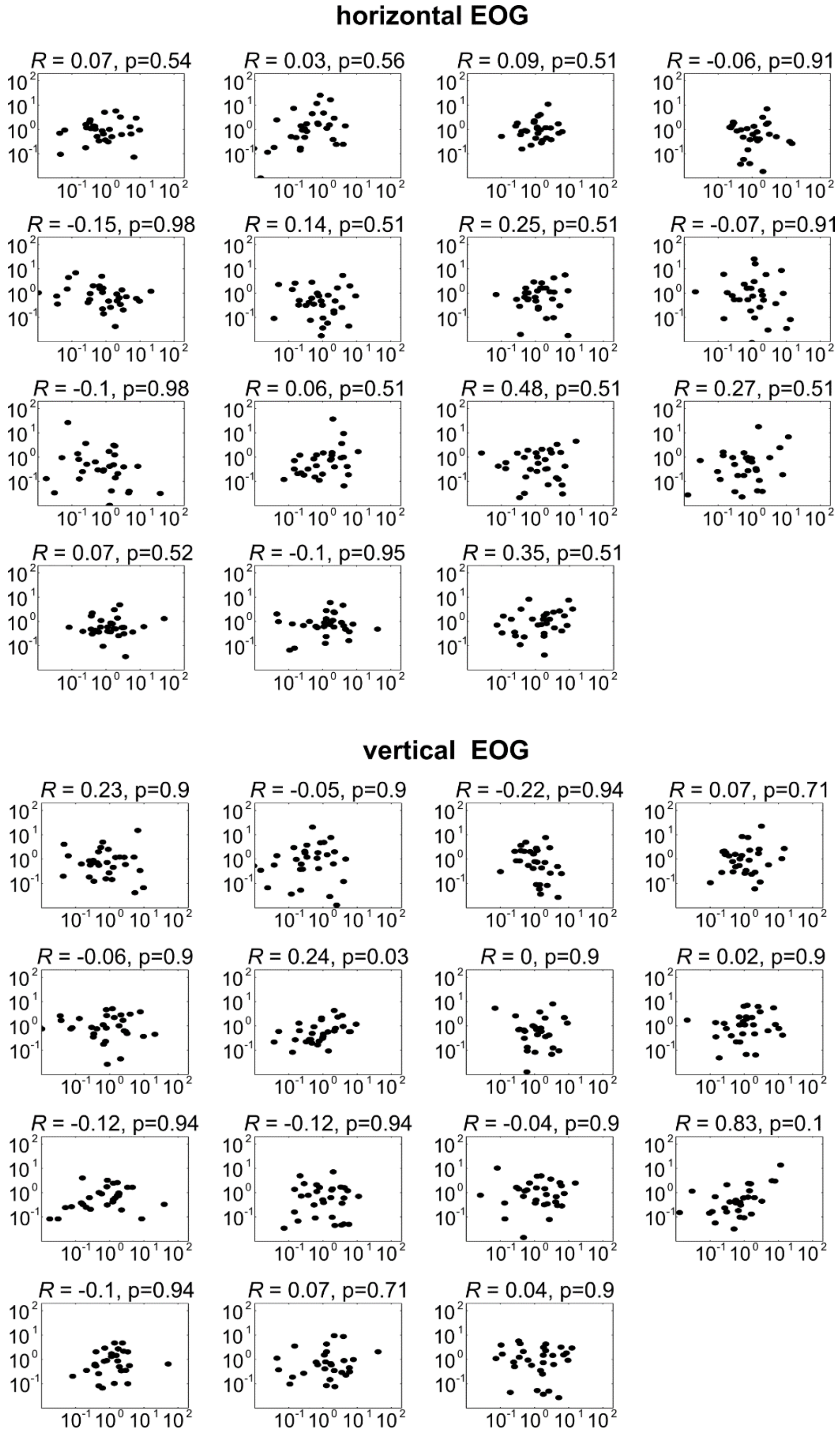

Supplementary Figure 1, Correlation between single-trial 1-Hz EOG power (x axis) and single-trial 1-Hz EEG power (y axis) in Experiment 1. Each plot shows data from one participant and each dot is data from a trial. The R- and P-values (bootstrap) are shown in the title of each plot. FDR correction is applied the P-values for all the 15 participants. No participant showed a significant correlation between EOG and EEG (even before the FDR correction).

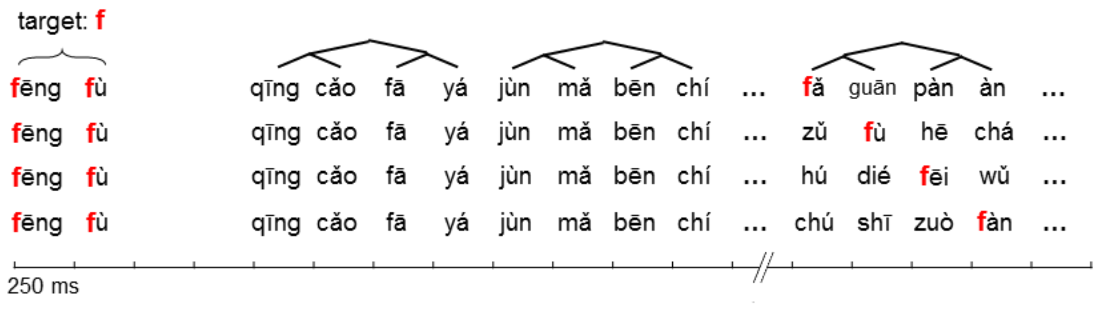

Supplementary Figure 2. Trial structure of Experiment 3. Four possible trials are illustrated. In each trial, a bisyllabic word is presented at the beginning and the both syllables in the word have the same initial phoneme (e.g., /f/ in the figure). That phoneme is the target phoneme the participants have to detect in the trial. One second after the bisyllabic word, a sequence of sentences (N = 11) are played and the target phoneme appears once in the sequence. It may appear as the 1st, 2nd, 3rd, or 4th syllable of a sentence (illustrated in the 4 rows).

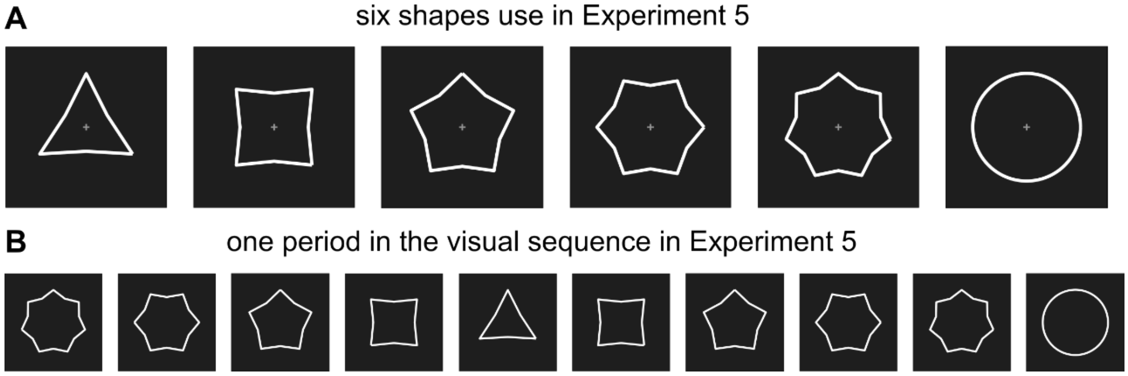

Supplementary Figure 3, shapes presented in Experiment 5. A) Six shapes were presented. One was a circle and the other 5 were adapted from regular polygons that had the same circumscribed circle. If the vertex coordinates of a regular *N*-sided polygon are (x_1,y_1), (x_2,y_2), …, and (x_N,y_N), the vertices of the adapted polygons are (x_1,y_1), $\alpha(x_1+x_2, y_1+y_2)$, (x_2,y_2), $\alpha(x_2+x_3, y_2+y_3)$, $(x_3,y_3)\ldots$, and (x_N,y_N), where $\alpha = 0.45$. In other words, the midpoint of each edge of a regular polygon was shrunk by 10%. In each period of the visual sequence, the circle and triangle-like shape appears once and other shapes appear twice. The visual target, i.e., a cross, is shown in each shape. B) The 6 shapes construct a 10-shape period that is repeated. In the 10-shape period, the triangle and circle are presented once and other shapes are presented twice.

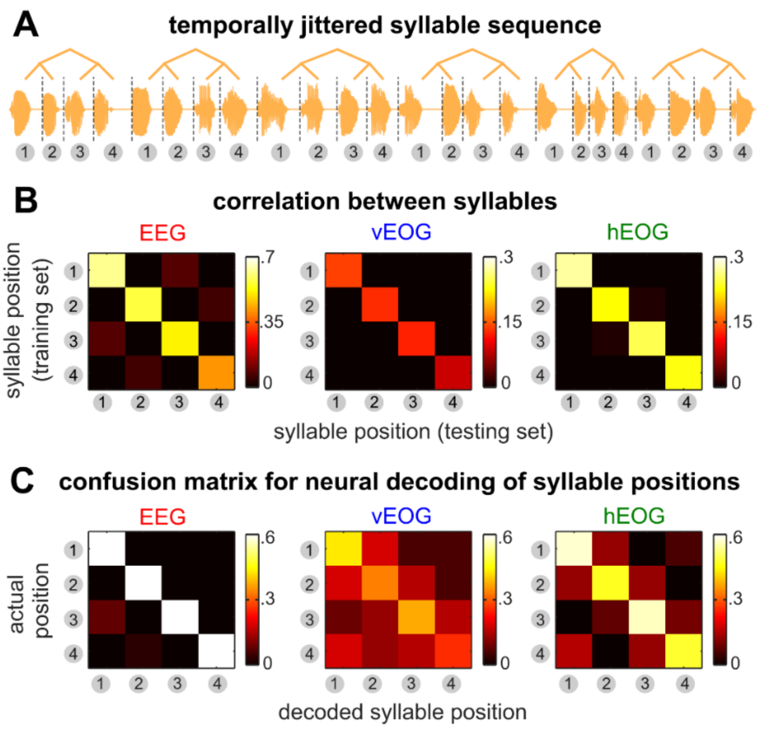

Supplementary Figure 4. EOG and EEG tracking of sentences for temporally jittered syllables in Experiment 6. A) Stimulus. The stimulus is a sequence of 4-syllable sentences and the stimulus onset asynchrony between syllables is randomized. B) Correlation between the responses to syllables in a testing sentence and the responses to syllables in training sentences. The diagonal structure indicates that syllables at the same position of a sentence have higher correlation. C) Confusion matrix for the decoding analysis. The position of each syllable in the testing sentence is decoded based on its correlation with syllables in the training sentences. Correctly decoded items locate in the diagonal of the confusion matrix.

2. Supplementary Tables

Supplementary Table 1. The statistical significance, mean, and SEM of spectral power for Experiment 1 (see also Fig. 1)[1].

P-value (mean/SEM)	1 Hz	2 Hz	4 Hz
EEG	0.1×10^{-3} (0.38/0.18)	0.1×10^{-3} (0.62/0.08)	0.1×10^{-3} (0.29/0.08)
vEOG	0.63×10^{-2} (8.73/4.07)	0.07 (1.7/1.46)	0.63×10^{-2} (0.44/0.25)
hEOG	0.27 (6.05/8.15)	0.6 (-0.08/0.43)	0.27 (0.04/0.04)

[1] The significance test is based on a bootstrap procedure comparing the power at the target frequency and the power averaged over 2 frequency bins right below the target frequency. For the mean and SEM, the power is normalized by subtracting the power averaged over 2 frequency bins right below the target frequency.

Supplementary Table 2. The statistical significance, mean, and SEM of spectral power for Experiment 2 (see also Fig. 2).

(i) Eyes Open, Moving Dots (sentence)			
P-value (mean/SEM)	1Hz	2Hz	4Hz
EEG	0.1×10^{-3} (0.45/0.12)	0.1×10^{-3} (0.57/0.09)	0.1×10^{-3} (0.34/0.06)
vEOG	0.03 (14.13/10.43)	0.3×10^{-3} (11.5/4.79)	0.19 (0.27/0.35)
hEOG	0.08 (0.35/0.22)	0.17 (0.04/0.03)	0.29 (0.69×10^{-2}/0.01)
blink	0.34×10^{-2} ($0.15\times10^{-3}/0.84\times10^{-4}$)	0.3×10^{-3} ($0.63\times10^{-4}/0.21\times10^{-4}$)	0.13 ($0.18\times10^{-5}/0.16\times10^{-5}$)
saccade	0.01 ($0.25\times10^{-5}/0.14\times10^{-5}$)	0.05 ($0.14\times10^{-5}/0.1\times10^{-5}$)	0.36×10^{-2} ($0.25\times10^{-5}/0.11\times10^{-5}$)
pupil	0.99 (-55.14/24.49)	0.99 (-2.97/7.82)	0.99 (-0.10/2.02)
**(ii) Eyes Open, Blank Screen (sentence)**			
P-value (mean/SEM)	1Hz	2Hz	4Hz
EEG	0.1×10^{-3} (0.38/0.09)	0.1×10^{-3} (0.38/0.07)	0.1×10^{-3} (0.41/0.07)
vEOG	0.02 (27.24/17.87)	0.02 (9.35/6.6)	0.12 (0.5/0.49)
hEOG	0.66 (-0.23/0.56)	0.38 (0.06/0.1)	0.3×10^{-3} (0.1/0.03)
blink	0.3×10^{-3} ($0.3\times10^{-3}/0.14\times10^{-3}$)	0.3×10^{-3} ($0.61\times10^{-4}/0.27\times10^{-4}$)	0.18 ($0.25\times10^{-5}/0.29\times10^{-5}$)
saccade	0.17 ($0.13\times10^{-5}/0.16\times10^{-5}$)	0.16 ($0.2\times10^{-5}/0.18\times10^{-5}$)	0.12 ($0.17\times10^{-5}/0.11\times10^{-5}$)
pupil	0.53 (-1.69/19.01)	0.53 (0.23/3.15)	0.15 (2.03/1.36)
**(iii) Eyes Closed (sentence)**			
P-value (mean/SEM)	1Hz	2Hz	4Hz
EEG	0.1×10^{-3} (0.49/0.12)	0.1×10^{-3} (0.33/0.1)	0.1×10^{-3} (0.42/0.09)
vEOG	0.01 (2.04/1.21)	0.06 (0.26/0.28)	0.3×10^{-3} (0.18/0.06)
hEOG	0.01 (3.36/1.96)	0.41 (0.02/0.09)	3.9×10^{-2} (0.05/0.02)
**(iv) Eyes Open, Moving Dots (control)**			
P-value (mean/SEM)	1Hz	2Hz	4Hz
EEG	0.59 (-0.63×10^{-2}/0.03)	0.31 (0.8×10^{-2}/0.01)	0.3×10^{-3} (0.17/0.04)
vEOG	0.36 (2.87/8.85)	0.36 (1.47/4.55)	0.36 (0.6/0.67)
hEOG	0.87 (-0.46/0.41)	0.57×10^{-2} (0.17/0.07)	0.09 (0.03/0.02)
blink	0.23 ($0.52\times10^{-4}/0.58\times10^{-4}$)	0.27 ($0.11\times10^{-4}/0.2\times10^{-4}$)	0.08 ($0.55\times10^{-5}/0.34\times10^{-5}$)
saccade	0.5 ($0.23\times10^{-8}/0.14\times10^{-5}$)	0.16 ($0.18\times10^{-5}/0.14\times10^{-5}$)	0.16 ($0.23\times10^{-5}/0.2\times10^{-5}$)
pupil	1 (-195.8/70.57)	0.17 (18.55/12.78)	0.57 (1.11/3.86)

Supplementary Table 3. The statistical significance, mean, and SEM of spectral power for Experiment 3 (see also Fig. 3).

**Attending to the 1st Syllable (Eyes Closed)**			
P-value (mean/SEM)	1Hz	2Hz	4Hz
EEG	0.1×10^{-3} (1.42/0.37)	0.1×10^{-3} (0.39/0.13)	0.1×10^{-3} (0.54/0.12)
vEOG	0.15×10^{-3} (30.69/10.76)	0.15×10^{-3} (5.29/2.35)	0.04 (0.41/0.33)
hEOG	0.3×10^{-3} (10.39/4.53)	0.07 (0.24/0.19)	0.57 (-0.7×10^{-2} /0.03)
**Attending to the 3rd Syllable (Eyes Closed)**			
P-value (mean/SEM)	1Hz	2Hz	4Hz
EEG	0.1×10^{-3} (1.13/0.5)	0.1×10^{-3} (0.37/0.12)	0.1×10^{-3} (0.68/0.18)
vEOG	0.1×10^{-3} (30.14/12.59)	0.1×10^{-3} (4.62/2.63)	0.1×10^{-3} (1.42/0.92)
hEOG	0.02 (8.03/7.78)	0.02 (0.49/0.34)	0.06 (0.04/0.03)

Supplementary Table 4. The statistical significance, mean, and SEM of spectral power for Experiment 4 (see also Fig. 4).

**Attending to the 1st Tone (Eyes Open)**			
P-value (mean/SEM)	1Hz	2Hz	4Hz
vEOG	0.03 (25.99/25.43)	0.51 (-0.1/2.41)	0.51 (0.05/0.24)
hEOG	0.11 (0.49/0.59)	0.52 (-0.28×10^{-2}/0.05)	0.02 (0.05/0.02)
blink	0.3×10^{-3} (0.16×10^{-3}/0.14×10^{-3})	0.81 (-0.1×10^{-4}/0.13×10^{-4})	0.81 (-0.69×10^{-6}/0.17×10^{-5})
saccade	0.1×10^{-3} (0.84×10^{-5}/0.41×10^{-5})	0.49 (0.95×10^{-7}/0.23×10^{-5})	0.84 (-0.14×10^{-5}/0.13×10^{-5})
pupil	0.06 (19.41/16.18)	0.08 (1.24/1.43)	0.9 (-0.27/0.25)
**Attending to the 3rd Tone (Eyes Open)**			
P-value (mean/SEM)	1Hz	2Hz	4Hz
vEOG	0.14 (11.18/12.37)	0.32 (1.44/2.49)	0.54 (-0.02/0.21)
hEOG	0.83 (-0.27/0.28)	0.83 (-0.02/0.08)	0.83 (-0.01/0.03)
Blink	0.02 (0.13×10^{-3}/0.98×10^{-4})	0.18 (0.11×10^{-4}/0.15×10^{-4})	0.17 (0.2×10^{-5}/0.2×10^{-5})
Saccade	0.02 (0.46×10^{-5}/0.39×10^{-5})	0.29 (0.62×10^{-6}/0.13×10^{-5})	0.02 (0.38×10^{-5}/0.29×10^{-5})
Pupil	0.21 (8.41/12.24)	0.06 (2.35/1.91)	0.11 (0.51/0.51)
**Attending to the 1st Tone (Eyes Closed)**			
P-value (mean/SEM)	1Hz	2Hz	4Hz
vEOG	0.15×10^{-3} (7.93/4)	0.15×10^{-3} (2.13/0.86)	0.4×10^{-3} (0.18/0.1)
hEOG	0.02 (2.26/1.36)	0.13 (0.15/0.22)	0.13 (0.05/0.05)
**Attending to the 3rd Tone (Eyes Closed)**			
P-value (mean/SEM)	1Hz	2Hz	4Hz
vEOG	0.1×10^{-3} (13.42/6.31)	0.1×10^{-3} (1.88/1.37)	0.1×10^{-3} (0.29/0.17)
hEOG	0.06 (2.51/2.32)	0.04 (0.37/0.3)	0.02 (0.07/0.04)

Supplementary Table 5. The statistical significance, mean, and SEM of spectral power for Experiment 4b (see also Fig. 5).

Attending to the 1st Tone (Eyes Open)			
P-value (mean/SEM)	1Hz	2Hz	4Hz
blink	0.3×10^{-3} ($0.26\times10^{-2}/0.17\times10^{-2}$)	0.9×10^{-2} ($0.65\times10^{-3}/0.71\times10^{-3}$)	0.8×10^{-2} ($0.79\times10^{-4}/0.56\times10^{-4}$)
saccade	0.46×10^{-2} ($0.12\times10^{-4}/0.53\times10^{-5}$)	0.15×10^{-2} ($0.46\times10^{-5}/0.23\times10^{-5}$)	0.15 ($0.12\times10^{-5}/0.13\times10^{-5}$)
pupil	0.18 (71.69/67.57)	0.79 (-24.38/35.21)	0.05 (8.31/5.54)
**Attending to the 5th Tone (Eyes Open)**			
P-value (mean/SEM)	1Hz	2Hz	4Hz
blink	0.15×10^{-3} ($0.23\times10^{-2}/0.13\times10^{-2}$)	0.15×10^{-3} ($0.18\times10^{-2}/0.11\times10^{-2}$)	0.02 ($0.59\times10^{-4}/0.48\times10^{-4}$)
saccade	0.18×10^{-2} ($0.91\times10^{-5}/0.41\times10^{-5}$)	0.24×10^{-2} ($0.55\times10^{-5}/0.32\times10^{-5}$)	0.01 ($0.33\times10^{-5}/0.16\times10^{-5}$)
pupil	0.29 (42.62/97.15)	0.06 (51.63/41.3)	0.11 (6.58/8.19)

Supplementary Table 6. The statistical significance, mean, and SEM of spectral power for Experiment 5 (see also Fig. 6).

Attending to the Triangle (Eyes Open)			
P-value (mean/SEM)	1Hz	2Hz	4Hz
blink	0.1×10^{-3} ($0.48\times10^{-2}/0.29\times10^{-2}$)	0.1×10^{-3} ($0.26\times10^{-2}/0.16\times10^{-2}$)	0.1×10^{-3} ($0.21\times10^{-3}/0.16\times10^{-3}$)
saccade	0.1×10^{-2} ($0.17\times10^{-4}/0.72\times10^{-5}$)	0.3×10^{-3} ($0.67\times10^{-5}/0.33\times10^{-5}$)	0.05 ($0.11\times10^{-5}/0.8\times10^{-6}$)
pupil	0.15×10^{-3} (330.5/138.8)	0.15×10^{-3} (90.3/33.1)	0.17 (1.35/1.73)
**Attending to the Circle (Eyes Open)**			
P-value (mean/SEM)	1Hz	2Hz	4Hz
blink	0.1×10^{-3} ($0.3\times10^{-2}/0.28\times10^{-2}$)	0.1×10^{-3} ($0.13\times10^{-2}/0.13\times10^{-2}$)	0.1×10^{-3} ($0.17\times10^{-3}/0.12\times10^{-3}$)
saccade	0.3×10^{-3} ($0.27\times10^{-4}/0.13\times10^{-4}$)	0.18×10^{-3} ($0.57\times10^{-5}/0.38\times10^{-5}$)	0.02 ($0.16\times10^{-5}/0.88\times10^{-6}$)
pupil	0.13×10^{-2} (254.94/160.09)	0.3×10^{-3} (52.95/15.97)	0.74 (-0.7/1.17)

Supplementary Table 7. The statistical significance, mean, and SEM of the EOG power change before/after the stimulus (see also Fig. 7).

**Experiment 1**				
P-value (mean/SEM)	vEOG (pre)	vEOG (post)	hEOG (pre)	hEOG (post)
eyes closed (sentence)	0.1×10^{-3}<br>(2781 /933)	0.1×10^{-3}<br>(2562 /740)	0.1×10^{-3}<br>(1549.07/738)	0.3×10^{-3}<br>(1022 /448)
**Experiment 2**				
P-value (mean/SEM)	vEOG (pre)	vEOG (post)	hEOG (pre)	hEOG (post)
moving dots (sentence)	0.2×10^{-3}<br>(2683 /804)	0.27×10^{-3}<br>(4600 /1181)	0.13×10^{-3}<br>(603 /96)	0.13×10^{-3}<br>(970 /141)
blank screen (sentence)	0.13×10^{-3}<br>(5426 /1510)	0.2×10^{-3}<br>(13312 /2215)	0.13×10^{-3}<br>(579 /182)	0.13×10^{-3}<br>(745 /267)
eye closed (sentence)	0.13×10^{-3}<br>(580 /239)	0.6×10^{-3}<br>(394 /161)	0.12<br>(163 /151)	0.21<br>(94 /124)
moving dots (syllables)	0.13×10^{-3}<br>(5341 /1437)	0.2×10^{-3}<br>(9361 /1908)	0.13×10^{-3}<br>(764 /185)	0.13×10^{-3}<br>(1195 /247)
**Experiment 3**				
P-value (mean/SEM)	vEOG (pre)	vEOG (post)	hEOG (pre)	hEOG (post)
eyes closed (syllable 1)	0.22×10^{-2}<br>(1666 /812)	0.5×10^{-3}<br>(2065 /880)	0.1×10^{-3}<br>(1558 /479)	0.1×10^{-3}<br>(1223 /456)
eyes closed (syllable 3)	0.2×10^{-3}<br>(1805 /887)	0.2×10^{-3}<br>(2803 /1199)	0.1×10^{-3}<br>(1311 /432)	0.1×10^{-3}<br>(1498 /483)
**Experiment 4**				
P-value (mean/SEM)	vEOG (pre)	vEOG (post)	hEOG (pre)	hEOG (post)
eyes open (tone1)	0.13×10^{-3}<br>(12599 /3323)	0.1×10^{-3}<br>(12085 /2901)	0.2×10^{-3}<br>(605 /311)	0.2×10^{-3}<br>(349 /176)
eyes open (tone3)	0.13×10^{-3}<br>(13516 /3325)	0.1×10^{-3}<br>(13366 /3039)	0.2×10^{-3}<br>(330 /188)	0.2×10^{-3}<br>(379 /167)
eyes closed (tone1)	0.13×10^{-3}<br>(611 /407)	0.1×10^{-3}<br>(431 /246)	0.96×10^{-2}<br>(289 /236)	0.43<br>(24 /116)
eyes closed (tone3)	0.5×10^{-3}<br>(561 /477)	0.1×10^{-3}<br>(717 /413)	0.96×10^{-2}<br>(324 /288)	0.28<br>(79 /137)

[2] The significance test is based on a bootstrap procedure comparing the pre/post-power with the power during the stimulus. For the mean and SEM, it is the difference between pre/post-stimulus power and the power during the stimulus.

Supplementary Table 8. The statistical significance, mean, and SEM of spectral power for Experiment 6 (see also Fig. 8).

	1Hz	2Hz	4Hz
EEG	0.15×10^{-3} (0.2/0.06)	0.27×10^{-2} (0.09/0.05)	0.15×10^{-3} (0.2/0.1)
vEOG	0.3×10^{-3} (0.27/0.04)	0.04 (0.01/0.89×10^{-2})	0.04 (0.38×10^{-2}/0.39×10^{-2})
hEOG	0.15×10^{-3} (0.11/0.04)	0.15×10^{-3} (0.54×10^{-2}/0.2×10^{-2})	0.18 (0.11×10^{-3}/0.15×10^{-3})

3. Supplementary Methods

Decoding Analysis

A decoder was employed to test if the EOG/EEG responses to the 4 syllables in a sentence show reliable differences. The EOG/EEG response to each syllable was extracted from 250 ms before to 750 ms after the syllable onset. The response to each syllable was baseline corrected based on the mean value in the 250-ms pre-stimulus interval averaged over all syllables. In the experiment, 90 trials of syllable sequences were presented and each trial contained 48 syllables. In the decoding analysis, the response to each of the 48 syllables were averaged across trials, and the responses to the first 4 syllables were excluded from further analysis to avoid the auditory onset response.

The decoding analysis followed a leave-one-out cross validation procedure. Each time, 40 syllables (from 10 sentences) were used as the training set and the remaining 4 syllables (from 1 sentence) was used as the testing set. The positions of the training syllables were labeled (i.e., the 1st, 2nd, 3rd, or 4th syllable in a sentence) while the position of the testing syllable was determined by the decoder. The decoder calculated the correlation between the response to each testing syllable and the response to each of the training syllables. On average, if the testing syllable was more correlated with the response to the kth syllable in the training sentences, it was decoded as the response to the kth syllable of the testing sentence.

A binomial test was employed to test if the number of trials being correctly decoded in the decoding analysis was significantly above chance. Since there were 4 possible positions in a sentence, chance level correct rate was 1/4 for each syllable. The experiment had 15 participants and each participant had 11 decoding results from the leave-one-out procedure. Therefore the chance level distribution of the number of correctly decoded trials was subject to a binomial distribution (p = 1/4 and N = 165).

Time-Warpping Analysis

Based on the neural response to non-isochronous syllable sequences, we simulated a neural response to isochronous syllable sequences. It was assumed that the same response was triggered by the jth syllable in a trial (j = 1, 2, …, 48), and therefore the response in the ith trial was formulated as the following:

$$s_i(t) = \Sigma_j\, h_j(t) * \delta(t - t_{i,j}) + e_i(t), \quad (1)$$

where $s_i(t)$ was the response in the ith trial, $h_j(t)$ was the response evoked by the jth syllable, $\delta(t)$ was the Dirac delta function, $t_{i,j}$ was the time when the jth syllable in the ith trial was presented, and $e_i(t)$ was the residual error that could not be explained by the current model. The response to each syllable, i.e., $h_j(t)$, was estimated based on all trials using a deconvolution procedure, i.e., normalized reverse correlation (Theunissen et al. 2001). The response to a 4-Hz syllable sequence was simulated using the following equation:

$$\hat{s}_i(t) = \Sigma_j\, h_j(t) * \delta(t - 0.25j), j = 5, 6, \ldots, 48 \quad (2)$$

In the simulation, the response to the first sentences in each trial was excluded to avoid the onset response.

Reference

Theunissen FE, David SV, Singh NC, Hsu A, Vinje, WE, Gallant JL. Estimating spatio-temporal receptive fields of auditory and visual neurons from their responses to natural stimuli. Network: Computation in Neural Systems 12, 289-316 (2001).

4. Supplementary Notes

We apply a decoding analysis to test if the EOG/EEG response to a syllable is significantly affected by the syllable's position within a sentence. In this analysis, the response to each syllable in a testing sentence is correlated with the responses to syllables in a set of training sentences, using a cross-validation procedure. If the response to a syllable is mostly correlated with the kth syllable (k = 1, 2, 3, or 4) of the training sentences, it is decoded as the response to the kth syllable of the testing sentence. The correlation matrix and the confusion matrix of the decoding results are shown in Supplementary Fig. 4B and 4C respectively. A diagonal structure can be seen from both matrices, demonstrating that the responses to the 4 syllables in a sentence can be dissociated based on their waveforms. The correct rate of the decoding analysis is 85%, 38%, and 53% for EEG, vertical EOG, and horizontal EOG respectively, all above chance ($P = 1 \times 10^{-5}$, 3×10^{-5}, and 2×10^{-15}, binomial test).
