# Supplementary material for: Eye activity tracks task-relevant structures during speech and auditory sequence perception
Source: Nat Commun. 2018 Dec 18;9:5374. doi: 10.1038/s41467-018-07773-y (PMC6299078; doi:10.1038/s41467-018-07773-y)
Supplement: Supplementary file 3 — Description of Additional Supplementary Files [file 41467_2018_7773_MOESM3_ESM.docx]

**Description of Supplementary Information Files**

**File Name:** Supplementary Data 1

**Description:** Data and the MATLAB scripts to analyze data and plot each figure are described below**:**

File Name: Figure1.m

Description: The MATLAB code to process data in Experiment 1 and plot the

results as displayed in Figure 1.

File Name: Figure2.m

Description: The MATLAB code to process data in Experiment 2 and plot the

results as displayed in Figure 2.

File Name: Figure3.m

Description: The MATLAB code to process data in Experiment 3 and plot the

results as displayed in Figure 3.

File Name: Figure4.m

Description: The MATLAB code to process data in Experiment 4 and plot the

results as displayed in Figure 4.

File Name: Figure5.m

Description: The MATLAB code to process data in Experiment 4b and plot the

results as displayed in Figure 5.

File Name: Figure6.m

Description: The MATLAB code to process data in Experiment 5 and plot the

results as displayed in Figure 6.

File Name: Figure7.m

Description: Calculate the stimulus-related power change for vertical and

horizontal EOG and plot the results as displayed in Figure 7.

File Name: Figure8.m

Description: The MATLAB code to process data in Experiment 6 and plot the

results as displayed in Figure 8.

File Name: Figure9.m

Description: The MATLAB code to process data in Experiments 1-5 and plot

results as displayed in figure 9.

Folder Name: tools

Description: MATLAB functions called by Figure1.m to Figure9.m.

Folder Name: Data

Description: The data recorded from Experiments 1-6. Detailed information is

show bellow:

File Name: ybE1.mat

Description: EEG data recorded in Experiment 1

File Name: yeE1.mat

Description: EOG data recorded in Experiment 1

File Name: ybE2.mat

Description: EEG data recorded in Experiment 2

File Name: yeE2.mat

Description: EOG data recorded in Experiment 2

File Name: etkE2.mat

Description: Eyetracking data recorded in Experiment 2

File Name: ybE3.mat

Description: EEG data recorded in Experiment 3

File Name: yeE3.mat

Description: EOG data recorded in Experiment 3

File Name: yeE4.mat

Description: EOG data recorded in Experiment 4

File Name: etkE4.mat

Description: Eyetracking data in Experiment 4

File Name: etkE4b.mat

Description: Eyetracking data in Experiment 4b

File Name: etkE5.mat

Description: Eyetracking data in Experiment 5

File Name: ERP-yb.mat

Description: Time-warpped EEG responses in Experiment 6.

File Name: TRF-yeH.mat

Description: Time-warpped vertical EOG responses in Experiment 6.

File Name: TRF-yeV.mat

Description: Time-warpped horizontal EOG responses in Experiment 6.
